# Transcriptome analysis indicates improved adipose tissue function in growing Iberian pig fed olive by-products based diets

**DOI:** 10.3389/fgene.2025.1571393

**Published:** 2025-05-16

**Authors:** Patricia Palma-Granados, Juan M. García-Casco, Ramón Peiró-Pastor, Cristina Óvilo, Miguel A. Delgado, Fabián García, Adrián López-García, Elena González, María Muñoz

**Affiliations:** ^1^ Centro de I+D en Cerdo Ibérico, Instituto Nacional de Investigación y Tecnología Agraria y Alimentaria (INIA-CSIC), Zafra, Spain; ^2^ Department Mejora Genética Animal, Instituto Nacional de Investigación y Tecnología Agraria y Alimentaria (INIA-CSIC), Madrid, Spain; ^3^ Department Producción Animal, Escuela de Ingenierías Agrarias, Universidad de Extremadura, Badajoz, Spain

**Keywords:** olive by-products, native pigs, Iberian pigs, transcriptome, alternative diets, backfat

## Abstract

Supplementing diets with olive by-products offers promising benefits for alleviating animal stress caused by feed restriction without compromising growth. The aim of this study was to explore the transcriptome of backfat in Iberian pigs fed experimental diets based in olive by-products. This study involved 15 pigs, which were placed into three diet groups: a Control (C) group, a dry olive pulp (DOP) group, which was fed a compound feed elaborated with olive pulp, and a wet crude olive cake (WCOC) group, which was fed a compound feed and an olive cake silage provided *ad libitum*. The pigs were fed these diets for 191 days, and at a body weight of 95 kg, backfat biopsies were taken, and transcriptome analyses were performed on 5 animals per group. Compared to the C group, we identified 411 and 924 differentially expressed genes (DEG; q < 0.05, |Fold Change|>1.5) for DOP and WCOC diets, respectively. In the DOP diet, functions related to *polysaccharides metabolism* were significantly activated, while the WCOC exhibited activated biological processes associated with *apoptosis* and *cellular death*. Both supplemented diets showed inhibition of functions involved in *inflammatory* and *immune responses*, as well as *reactive oxygen species production*. Furthermore, in the WCOC diet, functions related to *cholesterol* and *lipid metabolism* were repressed. In both comparisons, the *ADIPOQ* gene played a key role in the majority of affected functions. Our findings suggest that olive by-products may enhance adipose tissue function, which could have positive implications for animal health.

## 1 Introduction

The Iberian pig is a native breed from southwest Spain, traditionally raised using a free-range system that incorporates a unique fattening nutritional strategy named *Montanera*. This traditional method relies on the natural resources available in the environment, particularly acorns and pasture. Before entering this final fattening stage, the pigs undergo a period of feed restriction during their growth stage to attain the desired weight and body composition (Lopez-Bote, 1998). However, this process can lead to an undesirable stress for the animals, conflicting with principles of animal welfare.

The use of diets based on by-products from agricultural industries, such as flax, grapes and pumpkin (Vlaicu et al., 2019), as well as chestnuts and sugar beet (Díaz et al., 2009), which have low energy and high fiber content, could help minimize the stress caused by food restriction by increasing the feeling of satiety. Furthermore, their use in animal feeding should contribute to sustainability and the circular economy by reducing costs in agricultural and livestock production, as they are obtained from local products (de Miguel et al., 2015). Among the by-products available for animal feed, those from the olive industry can also enhance animal health and prevent diseases due to their high content of bioactive compounds, such as antioxidant molecules and essential fatty acids (Otero et al., 2021). On the other hand, the presence of dietary fiber in these by-products can positively influence the digestive health of the animals, supporting better intestinal function and nutrient absorption (Hu et al., 2023).

Differential gene expression studies using RNA-sequencing (RNA-seq) technology enable the identification of changes in the expression of genes involved in key biological processes and the exploration of pathways, mechanisms, and biological processes altered in different experimental groups. Consequently, numerous studies have investigated the effects of diverse diets on the transcriptome of pigs. For instance, Muñoz et al. (2021) observed alterations in the expression of genes related to lipid and cholesterol metabolism in the liver, as well as genes associated with growth, development, and meat quality in muscle, in Duroc × Iberian crossbred pigs fed low-protein diets. Benítez et al. (2017) observed differences in the expression levels of lipogenic and lipolytic genes between animals fed with high-oleic diets and those fed carbohydrate-rich diets. These same authors demonstrated that diets with different energy sources significantly affect biological pathways related to inflammation, lipid metabolism, and fat tissue development in the adipose tissue of Iberian pigs (Benítez et al., 2019).

In previous studies, our group demonstrated the usefulness of olive by-products in feeding growing Iberian pigs (40–100 kg) prior to *Montanera* with minor impact on growth rates and meat quality at 160 kg (García Casco et al., 2017; Palma-Granados et al., 2022). Moreover, a positive effect was observed on fatty acids profile, with an increase of oleic acid (C18:1) and a decrease of saturated fatty acids (SFA) (Palma-Granados et al., 2022). Additionally, our study revealed that the presentation format of these by-products, whether as pellets or as silage form, could differently affect the animal’s biology. In order to achieve a deeper understanding of gene regulation and biological processes affected by this type of feeding, the present nutrigenomics study analyzed the backfat transcriptome of Iberian pigs fed different diets based on olive by-products during their growing period.

## 2 Materials and methods

### 2.1 Olive by-products and diets

This study is part of a broader experiment on the overall effect of including olive by-products in the growing diets of Iberian pigs fattened in the *Montanera* system, a detailed description of the olive by-products and diets a has been previously explained in Palma-Granados et al. (2022).

In summary, two types of olive by-products were used in this work: dry olive pulp and wet crude olive cake, obtained at different stages of the normal olive oil extraction process. In the first step of this process, wet crude olive cake was obtained as a residue. This is a semi-liquid paste composed of olive pulp, skin, stone, and 71% water, making it one of the quickest, easiest, and most cost-effective by-products to obtain. Subsequently, the stone was removed, and a second oil extraction was conducted. The resulting mass was partially dehydrated to obtain dry olive pulp, which contains skin, pieces of olive stone, and a small proportion of olive oil (Molina-Alcaide and Yáñez-Ruiz, 2008).

**FIGURE 1:**
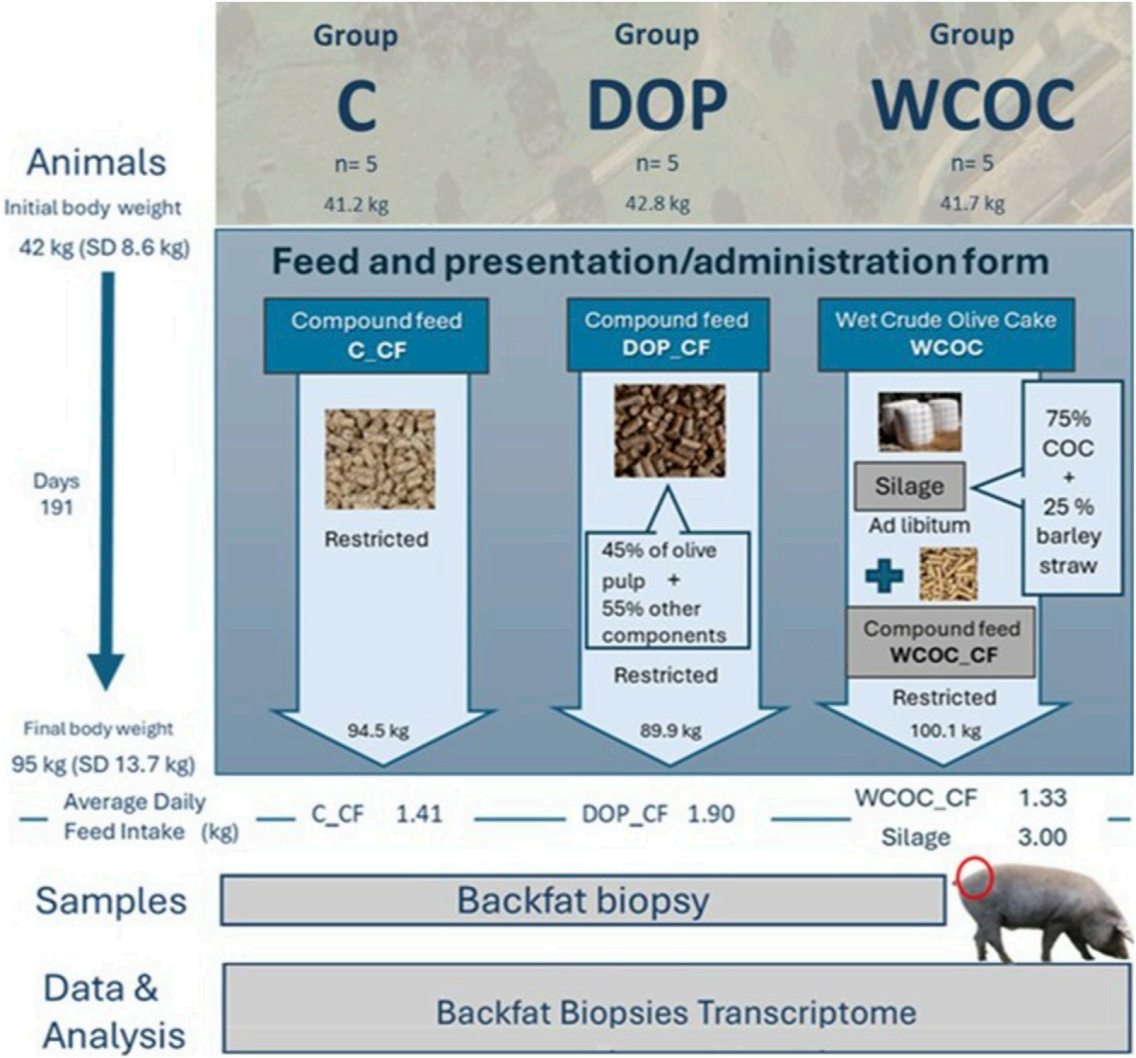
Description of the experimental design, olive by-products, and diets.

Three diets were employed. Control diet (C) consisted of a compound feed (C_CF) formulated to cover the protein and energy requirements of the growing period. A second diet (DOP) was based on a compound feed (DOP_CF) that contains 45% of olive pulp by-product in its composition. And a third diet (WCOC) included a compound feed specifically formulated for this group without by-product (WCOC_CF) and a silage with a mixture comprising 75% of crude olive cake and 25% of barley straw. The analytical compositions of by-products and the feed used in each dietary treatment are shown in Supplementary Table S1. The corresponding ingredients of the different compound feeds supplied to each group are described in Supplementary Table S2. In Figure 1, a description of by-products, diets and groups is presented.

### 2.2 Animal material

Animal manipulations were performed according to the Spanish Policy for Animal Protection RD 53/2013, which meets the European Union Directive 2010/63/EU about the protection of animals used in experimentation. The experimental protocol was approved by the Animal Welfare Section of the Extremadura region (no: EXP-20161004, ref.: SBC/mbr).

This study is part of a broader experiment on the overall effect of including olive by-products in the growing diets of Iberian pigs, fattened in the traditional system and slaughtered at a commercial body weight of 160 kg (Palma-Granados et al., 2022). The experimental design included a total of 44 Iberian pigs. At 6.5 months of age and 42 ± 8.6 kg of body weight, the animals were randomly divided into three groups and assigned to separate pens (one group per pen). All pens had a total area of 110 m^2^, with both open and covered sections, and were positioned adjacent to one another to ensure consistent water availability and spatial orientation. Each group received one of the three dietary treatments during the growing period: Control (C, n = 15), dry olive pulp (DOP, n = 14) and wet crude olive cake (WCOC, n = 15) group, respectively. All compound feeds were provided as pellets: DOP_CF and WCOC_CF at 60%, and C_CF at 70% of *ad libitum* energy intake. The silage offered in the WCOC diet was supplied *ad libitum*. A detailed explanation of the diets used and the feeding procedure is provided in Palma-Granados et al. (2022). The animals were fed these diets for 191 days, reaching 95 ± 13.7 kg of body weight at the end of the growing period at 12 months old. The experiment was conducted on a commercial farm without individual feeders, so individual feed intake was not measured. Instead, the average daily feed intake (ADFI) was calculated per group at the end of the experiment. In the C group, ADFI was 1.41 kg per day and pig. For the DOP group, ADFI was 1.9 kg per day and pig, and for the WCOC group, compound feed intake was 1.33 kg per day and pig, while silage intake was 3 kg per day and pig.

Subsequently, backfat biopsies were taken for transcriptome analysis, approximately 4–6 cm from the midline of the spine in the dorsal lumbar region, at a depth of about 2 cm. Samples were obtained using a cylindrical biopsy device with a sharpened edge of 0.5 cm in diameter under local anaesthesia with 2% lidocaine-HCI (Fresenius Kabi, Barcelona, Spain). Pigs were previously sedated by intramuscular injection of 2 mg/kg of body weight of azaperon (Stresnil, Ecuphar, Barcelona, Spain). After the biopsy, the area was sprayed with oxytretracycline (Veterin Tenicol; MSD Animal Health, Salamanca, Spain). All necessary measures were taken to prevent and alleviate discomfort of the animals during and after the process. Biopsy samples from the 44 pigs were placed in cryotubes, snap frozen in liquid nitrogen, and stored at −80°C until analysis. For the RNA-seq analysis, five samples per dietary treatment were selected (15 in total), according to the quantity and quality of the RNA as the main criterion.

### 2.3 RNA isolation, library construction and sequencing

Total RNA was extracted using the RiboPureTM of High Quality total RNA kit (Ambion, Austin, TX, United States) following the manufacturer’s recommendations. RNA was quantified with a NanoDrop device (NanoDrop Technologies, Wilmington, United States) and the integrity was assessed using the RNA Integrity Number (RIN) value from the Agilent 2100 Bioanalyzer device (Agilent technologies, Santa Clara, CA, United States). RIN values ranged between 6.6 and 8.2.

Library preparation and sequencing paired-end libraries were prepared using TruSeq SBS Kit v3 (Illumina, San Diego, CA, United States) for each sample. Multiplex sequencing of the libraries was carried out on a HiSeq 2000 sequence analyzer (Illumina, Inc.) with four samples per lane according to the manufacturer’s instructions at Centro Nacional de Análisis Genómico (CNAG, Barcelona, Spain), with a target depth sequencing of 40 million of reads. Paired-end reads of 76 bp were generated. The raw sequence data have been deposited in the Gene Expression Omnibus (GEO) expression database under the accession number: GSE264195.

### 2.4 Bioinformatics

Quality analyses were performed for the reads’ files using FastQC software (Babraham Bioinformatics, http://www.bioinformatics.babraham.ac.uk/projects/fastqc/). Quality assessment included measurements of sequence read lengths, base-coverage, nucleotide contributions, base ambiguities, quality scores, and identification of over-represented sequences. All the samples met the quality control parameters, exhibiting uniform length, 100% coverage across all bases, 29% of A and T, and 21% of G and C nucleotide contributions, 50% GC on base content and <0.1% overrepresented sequences, as average. Reads were then mapped against the pig reference genome (Sscrofa11.1) using HISAT2 (Kim et al., 2019), the alignment parameters used were those established by the default HISAT2, except for--rna-strandness, where it was specified that the reads come from a “strand-specific” RNA sequencing protocol. Given that the percentage of mapped reads exceeded 80%, no additional trimming or filtering of reads was deemed necessary. Subsequently, HTSeq-count (Anders et al., 2015) was used to merge reads based on overlapping paired-end reads, aiming to identify genes and to count the reads associated with these genes. Differential expression analyses were carried out using the DESeq2 package (Love et al., 2014) in the R environment (Team, 2015). Genes were considered as differentially expressed (DEGs) when the log2 fold change (log2FC) of the expression differences between the experimental diet (DOP or WCOC) and C group was |log2FC|≥ 0.58 for each comparison and a q-value <0.05.

### 2.5 RNA-sequencing validation by real-time quantitative PCR

RNA used in the RNA-seq study was employed to perform the technical validation of differential expression results by qPCR. Primer sequences for each gene were designed to cover different exons and ensure the amplification of the cDNA. To achieve this, Primer Select Software (DNASTAR, Wisconsin, United States) was employed, using available ENSEMBL sequences. Detailed information regarding the specific primers is provided in Supplementary Table S3. First-strand cDNA synthesis was conducted using random hexamers and Superscript III (Invitrogen, Life Technologies, Paisley, United Kingdom) in 20 µL of total volume containing 0.5 µg of total RNA and following the supplier’s instructions. RT-qPCR was performed for a set of 10 selected genes including *ATF3, CLU, CSF1R, HMGCR, KERA, KIT, LGALS3, OLR1, PCK1,* and *RUNX1* (Supplementary Table S3). The selection of the two most stable endogenous genes for data normalization in each comparison was performed by evaluating *GAPDH, ACTB, TBP, EEF2, PPIA* and *B2M* with the Genorm software (Vandesompele et al., 2002). Actin Beta (*ACTB*) and Peptidylprolyl Isomerase (*PPIA*) genes were used to normalize the expression data. The quantification of the transcripts was carried out in a LightCycler480 device (Roche, Basel, Switzerland) using SYBR Green Mix (Roche, Basel, Switzerland), following standard procedures. Each sample was run three times, and dissociation curves were acquired for every individual replicate. Confirmation of the specific amplification of the genes was established through the observation of single peaks in the dissociation curves. The PCR efficiency for each gene was determined using a standard curve calculation, employing four points of the cDNA serial dilutions. Pearson correlations were calculated between the expression values derived from the normalized counts generated by DESeq and the normalized RT-qPCR expression data. Additionally, the concordance correlation coefficient (CCC) (Miron et al., 2006) between the fold change values estimated from RNA-seq and RT-qPCR expression measures, was calculated.

### 2.6 *In silico* functional analyses

The Ingenuity Pathway Analysis (IPA) software (Ingenuity Systems, Qiagen, CA, United States) was used to functionally annotate the DEGs and to explore the enrichment of biological functions. Moreover, it facilitated the evaluation of the activation or inhibition of canonical pathways, along with the identification of potential regulators associated with the DEGs.

## 3 Results

### 3.1 Characterisation of the backfat transcriptome

The transcriptome of the 15 selected pigs was characterized through the RNA-seq technique. In the C, DOP and WCOC groups, a total of 37.6, 41.4 and 38.3 million raw reads were obtained, respectively. The average percentage of mapped reads was equal to 85.9% in the C, 87.2% in the DOP and 87.5% in the WCOC groups. Supplementary Table S4 provides a summary of the number of reads obtained and the percentage of mapped reads per sample. This study revealed that the dietary supplementation of pigs with olive by-products have an impact at the transcriptional level in subcutaneous fat, allowing the identification of 411 differentially expressed genes (DEGs) between the DOP and C diets (Supplementary Table S5), with 220 being upregulated (log_2_FC from 0.59 to 6.59) and 191 inhibited (log_2_FC from −0.58 to −4.97) in DOP compared to the C diet. In the comparison between the WCOC and C diets, there were 924 DEGs (Supplementary Table S6), 281 of them upregulated (log_2_FC from 0.58 to 21.73) and 643 inhibited (log_2_FC from −0.58 to −4.53) in WCOC. Table 1 shows the most relevant DEGs between each of the experimental diets (DOP or WCOC) and the C group. Notably, the two comparisons shared 145 DEGs, with the expression differences of these genes being in the same direction in both comparisons (up or downregulated in both experimental diets supplemented with olive by-products in comparison to C diet).

**TABLE 1:** Logarithm of fold change (log2FC), p-value and q-value corresponding to the most relevant differentially expressed genes between experimental diet (DOP or WCOC) and Control groups in the backfat samples of Iberian pigs.

Gene^a^	log2FC	p-value	padj
DOP vs. Control diet			
*SLIT1*	6.59	8.85 × 10^−4^	2.83 × 10^−2^
*GPX2*	5.36	1.18 × 10^−4^	8.42 × 10^−3^
*TNFRSF11B*	4.18	2.45 × 10^−6^	6.25 × 10^−4^
*MYBPH*	4.08	7.22 × 10^−5^	6.15 × 10^−3^
*ANKRD34A*	3.93	3.67 × 10^−4^	1.73 × 10^−2^
*MMP9*	−4.20	2.34 × 10^−7^	9.86 × 10^−5^
*OCSTAMP*	−4.28	1.76 × 10^−8^	2.36 × 10^−5^
*TM4SF19*	−4.40	4.21 × 10^−7^	1.56 × 10^−4^
*TREH*	−4.88	2.14 × 10^−7^	9.70 × 10^−5^
*SPP1*	−4.97	8.98 × 10^−10^	2.66 × 10^−6^
WCOC vs. Control diet			
*KRT27*	21.73	4.98 × 10^−9^	6.07 × 10^−7^
*FRMD1*	4.18	4.12 × 10^−11^	9.40 × 10^−9^
*IYD*	3.13	1.86 × 10^−5^	5.88 × 10^−4^
*FAM131C*	2.69	1.18 × 10^−7^	9.30 × 10^−6^
*RIPK4*	2.54	8.97 × 10^−6^	3.31 × 10^−4^
*TMEM215*	−3.35	3.80 × 10^−4^	5.78 × 10^−3^
*TM4SF19*	−3.40	2.69 × 10^−5^	7.67 × 10^−4^
*AMELY*	−3.44	6.13 × 10^−3^	4.48 × 10^−2^
*TREH*	−3.50	7.36 × 10^−5^	1.67 × 10^−3^
*ATP6V0D2*	−4.53	1.82 × 10^−7^	1.35 × 10^−5^

^a^ Genes with the highest log2FC, values are shown in the table.

### 3.2 RNA-sequencing validation by real-time quantitative PCR

The RNA-seq experiment was validated by quantifying the relative expression of a selection of 10 genes using RT-qPCR. All the Pearson correlations between both measures of genes expression were positive (ranged from 0.755 to 0.993) and significant (p-value <0.05; Supplementary Table S7). The obtained CCC value (0.98) demonstrates a substantial strength-of-agreement of the experiment, validating the global reproducibility of the RNA-seq results. In general, the significance of the differential expression obtained from both techniques was similar, considering a p-value <0.1 for RT-qPCR as significant, with just one exception in the WCOC vs. C comparison, for *OLR1* gene, which was not significant following the analysis by qPCR (p = 0.14), despite the high correlation observed between both techniques. Overall, the correlations between the two methods were strong, reinforcing the reliability of the results obtained. This is likely attributable to differences regarding accuracy, sensitivity and specificity of each method, but overall, RNA-seq results were consistent with those obtained from RT-qPCR.

### 3.3 Functional analyses *in silico*

Biological function analyses using IPA revealed a significant modulation of biological functions in both DOP and WCOC diet groups compared to the C group. In the DOP diet, five functions were significantly activated compared to the C group, including *Synthesis of polysaccharide*, *Synthesis of glycogen* and *Metabolism of polysaccharide* (Table 2; Supplementary Table S8). Conversely, a total of 87 biological functions were predicted to be inhibited in DOP diet, with 55 being related to inflammatory and immune response, including the *Immune response of cells*, *Inflammatory response, Phagocytosis* and *Production of reactive oxygen species*.

**TABLE 2:** The most relevant functional processes in the set of differentially expressed genes between experimental diet (DOP or WCOC) and control groups in backfat samples of Iberian pigs.

Functions annotation^a^	p-value	z-score	Molecules	Molecules
DOP vs. Control diet				
Synthesis of polysaccharide	1.23 × 10^−5^	2.796	20	*ADIPOQ, B3GNT4, BMP5, CHGA, CHST1, CSGALNACT1, DYRK2, EGF, GLCE, GRB10, GYG1, HGF, HS6ST2, IL1R2, IRS1, PPARGC1A, PPP1R3G, PRKAA2, PRKAG3, SPP1*
Synthesis of glycogen	1.37 × 10^−4^	2.596	10	*CHGA, DYRK2, EGF, GRB10, GYG1, IRS1, PPARGC1A, PPP1R3G, PRKAA2, PRKAG3*
Metabolism of polysaccharide	2.92 × 10^−8^	2.254	29	*ADAMTS7, ADIPOQ, ADRB1, B3GNT4, BMP5, CHGA, CHST1, CSGALNACT1, CTSB, DYRK2, EDNRB, EGF, GLCE, GRB10, GYG1, HGF, HS6ST2, IL1R2, IRS1, MMP12, PDK4, PFKM, PHKG1, PPARGC1A, PPP1R3G, PRKAA2, PRKAG3, PYGM, SPP1*
Communication of cells	8.08 × 10^−6^	2.736	61	*ADIPOQ, ADRA2A, ADRB1, AGTR1, ASB2, BANK1, BMX, C3, CCR1, CD48, CD9, CLEC5A, CLEC7A, DAPK2, DOK2, EDNRB, EGF, FCER1G, FGF16, GNAZ, GPNMB, GRB10, HCLS1, HGF, IRS1, ITGAM, ITGB1BP2, ITGB2, KIT, LAT2, LPXN, LRP8, MAPK12, MST1R, NCEH1, NDRG2, NFAM1, NOS2, NRXN1, P2RY11, PRKAA2, PRKAG3, RGS10, RGS13, RGS9, RPS6KA1, S100A6, S1PR3, SH3BP2, SH3GL2, SHC3, SUCNR1, TBXA2R*
Biosynthesis of cyclic nucleotides	4.16 × 10^−6^	2.123	19	*ADRA2A, ADRB1, AGTR1, ALDH2, EGF, FBN2, GIPR, GNAZ, HGF, NOS2, NT5E, P2RY11, P2RY2, PTGR2, RGS10, RGS13, SPP1, SUCNR1, TBXA2R*
Phagocytosis	4.08 × 10^−7^	−3.688	37	*ACTR3, ADIPOQ, C3, CAPG, CD48, CLEC7A, CYBA, CYBB, EGF, FCER1G, FERMT3, GRN, GSN, HGF, ITGAM, ITGB2, LGALS3, MSR1, NCKAP1L, PLEK, PRKAA2, PTX3, RAP1GAP, RPS6KA1, SH3BP2, SIGLEC1, SLC11A1, SOAT1, SPI1, SUCNR1, TGM2, TLE3, TLR9, TREM2, TYROBP, VAV1, VIM*
Infiltration by neutrophils	2.51 × 10^−5^	−2.627	20	*ADAM8, ADIPOQ, C3, CCR1, CTSB, FCER1G, HGF, ITGAM, ITGB2, LGALS3, MMP12, MMP9, NOS2, NT5E, PDK4, PTX3, SPP1, ST14, TLR9, VAV1*
Production of reactive oxygen species	7.63 × 10^−5^	−2.02	31	*ADIPOQ, AGTR1, AKAP1, ALDH2, C3, CLEC7A, CYBA, CYBB, EGF, GRN, GSN, HGF, HK2, ITGAM, ITGB2, MAOB, MSR1, NCF2, NFE2L3, NOS2, PPARGC1A, PRKAA2, S100A6, SH3GL2, SPI1, SPP1, TGM2, TLR9, TNFRSF11B, TYROBP, VAV1*
Inflammatory response	1.11 × 10^−14^	−3.028	76	*ADAM8, ADIPOQ, ADRA2A, AGTR1, AIF1, ALDH2, ANGPTL4, AQP9, BCL2A1, BCL6, C3, CAPG, CCR1, CD37, CD84, CD9, CHGA, CLEC5A, CLEC7A, CLU, CTSB, CTSS, CYBA, CYBB, DAPK2, EDNRB, EGF, FCER1G, FEM1A, GNAZ, GPR137B, GRN, GSN, HGF, HOXA9, IL7, ITGAM, ITGB2, KIT, KLKB1, LCP1, LGALS3, MAOB, MMP9, MSR1, MST1R, MYO1F, NCKAP1L, NDRG2, NKG7, NOS2, NT5E, OLR1, PDE4B, PDK4…*
Immune response of cells	2.19 × 10^−8^	−4.071	51	*ACTR3, ADIPOQ, BCL6, C3, CAPG, CD180, CD48, CLEC5A, CLEC7A, COCH, CTSB, CTSD, CTSS, CYBA, EGF, FCER1G, FERMT3, GRN, GSN, HGF, IL7, ITGAM, ITGB2, LGALS3, MSR1, MST1R, NCKAP1L, NOS2, PLEK, PRKAA2, PTX3, RAP1GAP, RPS6KA1, SH3BP2, SIGLEC1, SLC11A1, SOAT1, SPI1, SUCNR1, TCIRG1, TGM2, TLE3, TLR8, TLR9, TNFRSF11B, TREM2, TRIM63, TRIM7, TYROBP, VAV1, VIM*
WCOC vs. Control diet				
Organismal death	5.82 × 10^−12^	6.904	332	*ACACA, ACLY, ADAM19, ADCY7, ADGRF5, ADIPOQ, ADM, ADRB1, AHCY, AIFM1, AKAP12, ARAP3, ARHGEF37, ARHGEF39, ASPM, ASPN, ATF3, BCAT1, BCL2A1, BHLHE40, BIRC5, BLNK, BMAL1, BMP6, BRCA2, BUB1, BUB1B, C1QA, C1QB, C1QC, C3, C5, CA4, CARD10, CBS/LOC102724560, CCL11, CCNB1, CCNB2, CCNF, CCR1, CD163, CD200, CD22, CD274, CD44, CDC20, CDC25B, CDC6, CDK1, CDKN1A…*
Apoptosis	4.56 × 10^−10^	4.224	318	*AATK, ACACA, ACLY, ACO2, ACSL1, ACVR1C, ADAM8, ADIPOQ, ADM, ADRB1, AFAP1L2, AIF1, AIFM1, AIFM3, AKAP12, AQP3, ARHGAP18, ARSB, ASIC1, ASPN, ATAD2, ATF3, ATF5, ATP6V0D2, B4GALT5, BBS10, BCL2A1, BHLHE40, BIRC5, BLNK, BMP6, BRCA2, BTK, BUB1, C1QA, C3, C5, CA4, CADM1, CBS/LOC102724560, CCL11, CCNB1, CD200, CD22, CD274, CD44…*
Cell death of tumor cell lines	3.61 × 10^−9^	3.917	221	*AATK, ACACA, ACO2, ACSL1, ACVR1C, ADIPOQ, ADM, ADRB1, AIFM1, AKAP12, ARL4C, ARSB, ASPN, ATAD2, ATF3, ATF5, ATP5F1A, BCL2A1, BHLHE40, BIRC5, BLNK, BMP6, BRCA2, BTK, BUB1, BUB1B, C3, C5, CADM1, CCNB1, CD274, CD44, CD48, CDC20, CDC6, CDCA5, CDK1, CDKN1A, CDKN2C, CDT1, CENPE, CENPF, CENPL, CENPN, CKAP2, CKAP2L, CKS2, CLSPN, CLU, COL18A1, COL1A1, CRABP2, CREB5…*
Apoptosis of tumor cell lines	1.58 × 10^−8^	3.053	180	*AATK, ACACA, ACO2, ACVR1C, ADIPOQ, ADM, AIFM1, AKAP12, ARSB, ASPN, ATAD2, ATF3, ATF5, BCL2A1, BHLHE40, BIRC5, BLNK, BMP6, BRCA2, BTK, BUB1, C3, C5, CCNB1, CD274, CD44, CD48, CDC20, CDC6, CDK1, CDKN1A, CDKN2C, CENPE, CENPF, CENPL, CENPN, CKAP2, CKS2, CLU, COL18A1, COL1A1, CRABP2, CREB5, CSF1R, CSTB, CTH, CTSB, CYCS, CYP1B1, DCLK1, DEPDC1, DLL1, DLL4, DUSP4, E2F1*
Necrosis	2.37 × 10^−11^	2.963	338	*AATK, ACACA, ACLY, ACO2, ACSL1, ACVR1C, ADAM8, ADIPOQ, ADM, ADRB1, AFAP1L2, AIFM1, AIFM3, AKAP12, ARHGAP18, ARL4C, ARSB, ASPN, ATAD2, ATF3, ATF5, ATP5F1A, ATP6V1B2, B4GALT5, BCL2A1, BHLHE40, BIRC5, BLNK, BMP6, BRCA2, BTK, BUB1, BUB1B, C1QA, C3, C5, C7, CA4, CADM1, CBS/LOC102724560, CCL11, CCNB1, CD200, CD22, CD274, CD300LB, CD44, CD48, CD86, CDC20, CDC25B, CDC6*
Synthesis of reactive oxygen species	9.18 × 10^−7^	−2.072	79	*ABCD1, ABCD2, ADIPOQ, AIFM1, ARAP3, ASIC1, C3, C5, CBS/LOC102724560, CCL11, CD44, CDKN1A, CLEC7A, CLU, COL18A1, CSTB, CTH, CXCL14, CYBB, CYCS, CYP1A1, CYP1B1, CYP2D6, DUOX2, E2F1, EZH2, F2, FN1, G6PD, GAB2, GRN, HGF, HK1, HK2, HSPB1, ITGAM, ITGB2, LEPR, LGALS3, MLPH, MPV17L, MRC1, MSR1*
Synthesis of fatty acid	1.33 × 10^−8^	−2.112	57	*ACACA, ACLY, ACOT7, ACSL1, ACSS2, ADIPOQ, BMP6, C1QTNF3, C5, CADM1, CCL11, CD209, CLEC7A, CLU, COTL1, CYP1A1, CYP1B1, CYP2D6, CYP7A1, DCLK1, ELOVL6, F2, FASN, FCER1G, FFAR4, FN1, G6PD, GIPR, GRB14, HGF, HNF4A, IL15, INSIG1, KIT, LDLR, LEPR, MID1IP1, NOX5, NTN1, OLR1, P2RY12, PDHB, PTGER2, PTPN7, RASGRP4, RUNX1, S1PR3, SCD, SEMA3A, SLC1A1, SLC25A1, SLC2A4, SLC45A3*
Synthesis of lipid	3.84 × 10^−5^	−2.630	102	*ACACA, ACAT2, ACLY, ACO2, ACOT7, ACSL1, ACSS2, ADGRF5, ADIPOQ, ADM, APOM, ATF3, BCO1, BHLHE40, BMAL1, BMP6, C1QTNF3, C3, C5, CADM1, CCL11, CD209, CLEC7A, CLU, COTL1, CXCL10, CYP1A1, CYP1B1, CYP2D6, CYP7A1, DCLK1, DHRS9, EBP, ELOVL6, F2, FABP3, FASN, FCER1G, FFAR4, FN1, G6PD, GIPR, GLA, GRB14, HGF, HMGCR, HMGCS1, HNF4A, HSD11B1, HSPA8, IDI1, IGFBP4, IL15, INSIG1*
Inflammatory response	5.24 × 10^−13^	−2.702	126	*ABCD1, ABCD2, ADAM8, ADGRF5, ADIPOQ, ADM, AFAP1L2, AIF1, AQP9, ARAP3, ATF3, BCL2A1, BLNK, BTK, C1QA, C1QTNF12, C1QTNF3, C3, C5, CAPG, CCL11, CCL26, CCR1, CD163, CD200, CD274, CD44, CD84, CDKN1A, CLEC5A, CLEC7A, CLU, CMKLR1, COL18A1, CSF1R, CTSB, CTSS, CXCL10, CXCL14, CYBB, E2F2, ECM1, EPHB1, F2, FANCA, FANCD2, FASN, FCER1G, FFAR4, FN1, GNAZ, GRN, HGF, HNF4A*
Immune response of cells	2.46 × 10^−5^	−4.692	79	*ACTR3, ADIPOQ, BHLHE40, BIRC5, BTK, C1QA, C3, C5, CAPG, CCL11, CD180, CD200, CD22, CD274, CD44, CD48, CD86, CDKN1A, CLEC5A, CLEC7A, COLEC12, CSF1R, CTSB, CTSS, CXCL10, CYP1B1, CYP2S1, FCER1G, FERMT3, FFAR4, FN1, GAB2, GRN, HGF, HSPA8, HSPB1, IL15, IL27RA, IL2RG, ISG15, ITGAM, ITGB2, KCNK13, KLF2, LDLR, LEPR, LGALS3, LUM, MRC1, MSR1, NCKAP1L, NFIL3, NLRP3, NTN1*

^a^ The ten biological functions were selected according to their biological relevance.

In the WCOC diet, eight significantly enriched functions were activated (Table 2; Supplementary Table S10). Among these, six functions were associated with apoptosis and cellular death. Additionally, 90 biological functions were predicted to be inhibited (Supplementary Table S10). Similar to the comparison between DOP and C pigs, functions associated to the immune and inflammatory systems were inhibited in the WCOC diet. Other inhibited functions included those affecting lipid metabolism, such as *Synthesis of Lipid, Synthesis of Fatty Acids*, *Fatty Acid Metabolism* and *Synthesis of ROS*.

The canonical pathway analysis using IPA agrees with the functional biological analyses in both diet comparisons. In the comparison with C group, two pathways were significantly activated in DOP: the *Apelin Adipocyte Signaling Pathways* and *AMP-activated Protein Kinase (AMPK) Signaling* (Table 3; Supplementary Table S9). Conversely, two pathways were significantly inhibited: *Phagosome Formation* and *Role of Osteoblast in Rheumatoid Arthritis Signaling*. These findings are consistent with the functional alterations related to metabolism and immune response observed in the DOP group. In contrast, in the comparison between the WCOC and C diets, no canonical pathways were significantly activated (Table 3; Supplementary Table S11). However, 16 pathways were significantly inhibited in WCOC, particularly those related to fatty acid metabolism and the cholesterol pathway, such as *Mevalonate Pathway I*, *Superpathway of Geranylgeranyldiphosphate Biosynthesis I* and *Superpathway of Cholesterol Biosynthesis* (Table 3). Additionally, several biological functions related to inflammation and immune response is also evident in the significant inhibition of canonical pathways. These pathways include *Phagosome formation*, *Pyroptosis Signaling pathway*, *Pathogen-Induced Cytokine Storm Signaling pathway*, and *Role of Pattern Recognition Receptors in Recognition of Bacteria and Viruses*.

**TABLE 3:** Canonical pathways with predicted direction in the set of differentially expressed genes between experimental diet (DOP or WCOC) and Control groups in backfat samples of Iberian pigs.

Ingenuity canonical pathways	p-value	z-score	Molecules
DOP vs. Control diet			
AMPK Signaling	1.91 × 10^−3^	2.714	*ADIPOQ, ADRA2A, ADRB1, CPT1C, GNAZ, HMGCR, IRS1, MAPK12, PFKM, PPARGC1A, PRKAA2, PRKAB2, PRKAG3*
Apelin adipocyte signaling pathway	3.90 × 10^−5^	2.646	*CYBB, GPX2, MAPK12, NCF2, PPARGC1A, PRKAA2, PRKAB2, PRKAG3*
Role of osteoblasts in rheumatoid arthritis signaling pathway	1.85 × 10^−2^	−2.530	*CTSB, CTSD, CTSH, CTSS, CTSZ, FRZB, FZD7, MMP12, MMP9, TNFRSF11B*
Phagosome formation	6.82 × 10^−5^	−2.746	*ACTR3, ADRA2A, ADRB1, AGTR1, AP1S3, C3, CCR1, CLEC7A, EDNRB, FCER1G, FZD7, GIPR, GPR137B, ITGAM, ITGB2, MAPK12, MC5R, MSR1, P2RY11, P2RY2, S1PR3, SUCNR1, TBXA2R, TLR8, TLR9, VAV1*
WCOC vs. Control diet			
Gustation pathway	1.63 × 10^−3^	−2	*ADCY7, ADCY8, ASIC1, FFAR4, KCNN2, KCNN4, LIPG, P2RX5, P2RY11, P2RY12, P2RY2, PRKAR2B, SCN8A, SLC2A4, TAS1R3, TRPM5*
Ethanol degradation IV	5.93 × 10^−3^	−2	*ACSL1, ACSS2, ALDH4A1, GPX7*
Ethanol degradation II	3.46 × 10^−2^	−2	*ACSL1, ACSS2, ALDH4A1, DHRS9*
Role of pattern recognition receptors in recognition of bacteria and viruses	3.13 × 10^−3^	−2.121	*C1QA, C1QB, C1QC, C3, C5, CLEC7A, IL15, IL17D, NLRP3, TLR2, TLR8, TLR9, TNFSF8*
Pyroptosis signaling pathway	3.71 × 10^−2^	−2.121	*GSDMB, NLRP3, PRKAR2B, PTGER4, PYCARD, TLR2, TLR8, TLR9*
Tumor microenvironment pathway	3.13 × 10^−3^	−2.183	*CD274, CD44, COL1A1, COL1A2, FGF18, FN1, HGF, LEPR, MMP16, MMP27, MMP9, MRAS, PDCD1LG2, SLC2A4, SPP1, TIAM1, TNC*
Mevalonate pathway I	4.99 × 10^−6^	−2.236	*ACAT2, HMGCR, HMGCS1, IDI1, MVD*
Superpathway of geranylgeranyldiphosphate biosynthesis I (via mevalonate)	7.08 × 10^−5^	−2.236	*ACAT2, HMGCR, HMGCS1, IDI1, MVD*
DNA methylation and transcriptional repression signaling	2.61 × 10^−2^	−2.333	*CDK1, CDK13, CHD5, E2F1, E2F2, E2F7, E2F8, HNF4A, HOXB3, TCF19, UHRF1*
Superpathway of cholesterol biosynthesis	2.88 × 10^−4^	−2.449	*ACAT2, EBP, HMGCR, HMGCS1, IDI1, MVD*
Kinetochore metaphase signaling pathway	4.22 × 10^−11^	−2.558	*BIRC5, BUB1, BUB1B, CCNB1, CDC20, CDCA8, CDK1, CENPA, CENPE, CENPL, CENPN, CENPT, CENPU, DNAH10, ESPL1, KIF2C, KNTC1, MAD2L1, MASTL, MXD3, NDC80, NEK2, NUF2, PLK1, PPP1R3C, SKA1, SPC24, TTK*
Phagosome formation	1.01 × 10^−4^	−2.887	*ACTR3, ADGRA3, ADGRB3, ADGRF5, ADRB1, APBB1IP, C3, CCR1, CD209, CLEC7A, CMKLR1, CMKLR2, COLEC12, FCER1G, FFAR4, FN1, FZD8, GAB2, GIPR, GPR153, GPR34, GPRC5A, ITGA11, ITGA8, ITGAM, ITGB2, MRAS, MRC1, MRC2, MSR1, OPRD1, P2RY11, P2RY12, P2RY2, PTGER2, PTGER4, S1PR3, SCARA3, SUCNR1, TAS1R3, TBXA2R, TIMD4, TLN2, TLR2, TLR8, TLR9, VAV1, XCR1*
Cell cycle control of chromosomal replication	5.29 × 10^−6^	−3.317	*CDC6, CDK1, CDK13, CDT1, MCM2, MCM3, MCM4, MCM6, ORC1, POLE, TOP2A*
Multiple sclerosis signaling pathway	5.23 × 10^−5^	−3.441	*AIFM1, ASIC1, C1QA, C1QB, C1QC, C3, C5, C7, EZH2, IL15, IL17D, MASP1, MMP9, NLRP3, PYCARD, TLR2, TLR8, TLR9, TNFSF8*
Role of osteoclasts in rheumatoid arthritis signaling pathway	4.97 × 10^−2^	−3.578	*ACP5, ADAM19, ADAM2, ADAM8, BLNK, BTK, COL16A1, COL18A1, COL1A1, COL1A2, COL21A1, CREB5, CSF1R, IKBKE, MMP16, MMP27, MMP9, MRAS, SFRP2, SFRP4, SHC3, SPI1, SPP1, TREM2, TYROBP*
Pathogen induced cytokine storm signaling pathway	1.51 × 10^−6^	−4.017	*BHLHE40, C3, C5, CCL11, CCL26, CCR1, CD163, CGAS, CLEC7A, COL16A1, COL18A1, COL1A1, COL1A2, COL21A1, CXCL10, CXCL14, HMGCR, IL15, IL17D, NLRP3, PYCARD, SLC2A4, SLC2A5, SPI1, SRGN, STX11, TLR2, TLR8, TLR9, TNFSF8*

## 4 Discussion

The experimental diets of this study are based on by-products of the olive oil industry: one diet incorporates olive pulp, while the other includes crude olive cake. While the use of olive pulp is more convenient in terms of handling, as it can be used as a compound feed, the drying process can increase the economic cost. A wet cake diet mixed with straw can facilitate *ad libitum* administration and increase fiber content. However, its high water content makes it impossible to include it as a compound feed. The use of these diets comes with certain limitations that must be comprehensively addressed. One of these limitations arises from the fact that dry and wet by-products have different nutritional compositions, requiring different ingredients in the formulation of the final diet, such as barley in the DOP diet and cereal straw in the WCOC diet. Despite efforts to adjust nutrients when incorporating by-products into pig diets, it is crucial to consider practical objectives and minimize the processing of these products. Formulating diets with specific compositions may result in additional costs which goes against the supposed inherent cost-effectiveness of using by-products. The current study, beyond examining the effect of an isolated nutrient on the animal, aimed to understand the underlying mechanisms of using these by-products in diets, considering that their overall composition in terms of protein, fiber, fat, and energy is variable. The impact of these olive by-product diets on growth performance, carcass composition, and meat quality traits were evaluated and reported by Palma-Granados et al. (2022), revealing only minor effects overall. Despite differences in feeding levels, restricted in the DOP and C groups, and *ad libitum* access to silage in the WCOC group, no significant differences in growth rate were observed among the groups (Palma-Granados et al., 2022). Only slight alterations in the fatty acid composition of backfat were detected. Pigs fed the DOP and WCOC diets exhibited lower levels of major SFA compared to the C group, along with higher concentrations of unsaturated fatty acids. Specifically, DOP pigs showed a general increase in unsaturated fatty acids, whereas WCOC pigs displayed elevated levels of polyunsaturated fatty acids (PUFA) (Palma-Granados et al., 2022).

**FIGURE 2:**
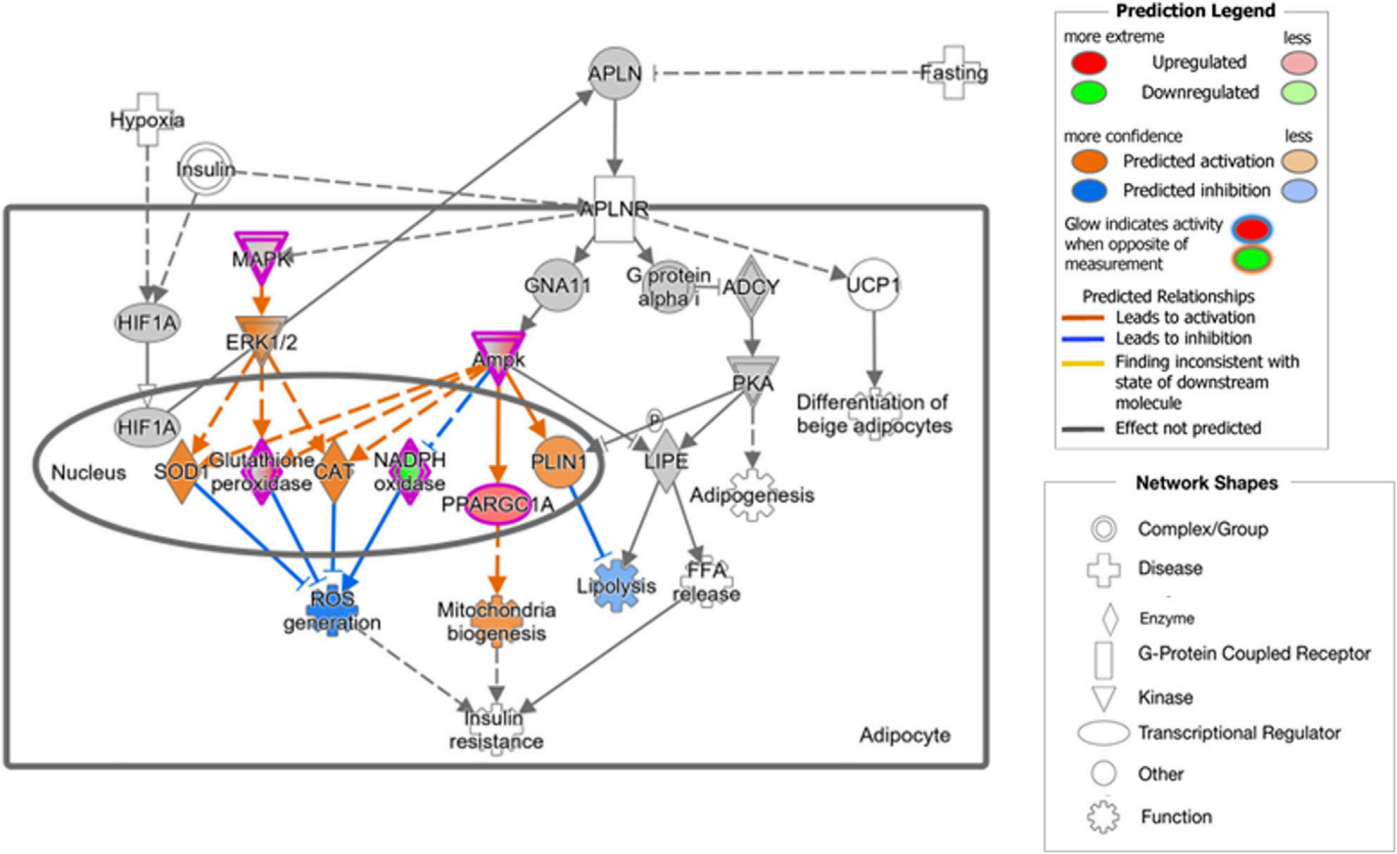
Biological processes and genes involved in the Apelin Adipocyte Signaling Pathway, significantly activated by the DOP diet. © 2020-2022 QIAGEN. All rights reserved.

Functional interpretation of the transcriptome results suggests that olive by-products seem to have significant effects on key biological functions, particularly carbohydrate and lipid metabolism. Specifically, the DOP diet may activate carbohydrate metabolism by promoting processes such as *Synthesis of polysaccharide*, *Synthesis of glycogen* and *Metabolism of polysaccharide*. This effect was accompanied by the overexpression of key genes involved in energy metabolism regulation, such as *Protein Kinase AMP-Activated Non-Catalytic Subunit Alpha 2* (*PRKAA2*), the *Non-Catalytic Gamma 3* (*PRKAG3*) and *Beta 2* (*PRKAB2*) *Subunits*, which encode subunits of the AMP-activated protein kinase (AMPK) heterotrimer. Activation of AMPK triggers the induction of peroxisome proliferator-activated receptor gamma coactivator 1 alpha (PPARGC1A), a key regulator of mitochondrial biogenesis and gluconeogenesis (Leick et al., 2010), which was also overexpressed in the DOP diet. Additionally, genes like *Glycogenin 1* (*GYG1*) and *Insulin Receptor Substrate 1* (*IRS1*) responsible for glycogen synthesis and the regulation of glucose metabolism, were also upregulated in the DOP diet. In addition, the DOP diet also induced the expression of *Adiponectin* (*ADIPOQ*) gene, which encodes a hormone secreted by white adipose tissue that plays a crucial role in energy homeostasis and in the regulation of glucose and lipid metabolism (Stefan and Stumvoll, 2002). Adiponectin can activate AMPK and peroxisome-proliferator-activated receptor α (PPAR-α) ligand functions. Upon activation, AMPK phosphorylates and inactivates acetyl-CoA carboxylase (ACACA) and beta-hydroxy beta-methylglutaryl-CoA reductase (Zhou et al., 2001), key enzymes regulating *de novo* biosynthesis of fatty acids and cholesterol, promoting fatty-acid combustion, glucose uptake, and lactate production. Through PPAR-α, adiponectin increases fatty-acid and energy consumption, reducing triglyceride content and enhancing insulin sensitivity in the liver and skeletal muscle (Yamauchi et al., 2002). It has been described that *ADIPOQ* gene is less expressed in Casertana pigs that in Large White pigs, with Casertana breed showing a greater fat accumulation (De Rosa et al., 2013). This finding agrees with the fact that these DOP animals, with higher expression of *ADIPOQ*, exhibited reduced backfat depth at both the 10th and last ribs compared to the C group (Palma-Granados et al., 2022). Moreover, by binding to its receptors, adiponectin enhances insulin sensitivity, and is downregulated in individuals with obesity and insulin resistance (Lihn et al., 2005). Studies have shown that high-fiber diets, such as the DOP diet, improve insulin resistance and glucose regulation (Weickert and Pfeiffer, 2018). Sánchez et al. (2012) showed that adiponectin levels increase with fiber intake, suggesting that fiber may act as an activator of *ADIPOQ* expression in the adipose tissue. Furthermore, supplementation with antioxidants, such as hydroxytyrosol and oleic acid, has been found to increase adiponectin levels (Scoditti et al., 2015). These findings are further supported by the activation of significant canonical pathways observed in the comparison between the DOP and C groups, including *AMPK Signaling*. Additionally, the *Apelin Adipocyte Signaling Pathway*, which leads to a decrease in lipolysis while promoting mitochondrial biogenesis (Figure 2), appear to be activated by the inclusion of dried pulp in the diet. Apelin, an adipocytokine secreted by adipocytes, acts as a ligand for the G protein-coupled receptor angiotensin receptor-like 1 (Fibbi et al., 2023) and its activation produces a decrease in the synthesis and release of fatty acids, promotion of brown fat over white fat, and interaction with other signaling pathways (Than et al., 2015). While the IPA analyses did not reveal significant impacts on biological processes directly associated with lipid metabolism, the activation of Apelin and AMPK signalling could potentially explain the results observed in the fatty acid profile of the adipose tissue in the DOP animals, which showed a decrease in SFA and an increase in PUFA compared to C pigs (Palma-Granados et al., 2022).

In contrast to the DOP diet, the WCOC diet did not impact carbohydrate metabolism, but it did significantly inhibit several functions (*Synthesis of Lipid, Synthesis of Fatty Acids* and *Fatty Acid Metabolism)* and canonical pathways (*Mevalonate Pathway I*, *Geranylgeranyldiphosphate Biosynthesis I* and *Cholesterol Biosynthesis*) associated with lipid metabolism. In addition, genes involved in the biosynthesis of fatty acids, including *Fatty Acid synthase* (*FASN*) and *ACACA*, were downregulated in the WCOC diet, alongside genes encoding enzymes responsible for fatty acid elongation and unsaturation, such as fatty acid elongase 6 (ELOVL6) and stearoyl-CoA desaturase (SCD). The inhibition of *SCD* gene might be influenced by the higher levels of C18:1 and C18:2 provided by the WCOC diet, as both regulate SCD activity (Ntambi, 1999; Smith et al., 2002). The repression of lipogenic genes and functions in WCOC pigs may explain the lower SFA proportions and higher PUFA levels in the subcutaneous tissue observed in this group compared to the C animals, despite both groups showing similar backfat thickness (Palma-Granados et al., 2022). Additionally, genes related to cholesterol metabolism, such as *Oxidized Low Density Lipoprotein Receptor 1* (*OLR1*), *Low Density Lipoprotein Receptor* (*LDLR*) and *Sterol O-Acyltransferase 1* (*SOAT1*) genes, were also repressed. These genes participate in the uptake of lipoproteins and the conversion of cholesterol into cholesterol esters, which are implicated in the transport and storage of lipids. Although cholesterol levels were not measured in this experiment, another study showed no significant effects on total cholesterol levels of Iberian pigs fed with olive by-products (Sánchez et al., 2022). This effect on cholesterol is also reflected in the several canonical pathways where Acetyl CoA is converted into Mevalonate through several reactions involving genes such as *Acetyl-CoA Acetyltransferase 2* (*ACAT2), 3-Hydroxy-3-Methylglutaryl-CoA Reductase (HMGCR),* and *Mevalonate Diphospho Decarboxylase (MVD),* all of which were downregulated in the WCOC diet (Table 3). The differential intake of nutrients from these olive by-products diets likely contributed to these effects, which aligns with studies showing that high-fiber diets regulate lipid metabolism. In fact, the cholesterol-lowering effects of soluble dietary fiber are well-documented (Surampudi et al., 2016) and our findings are consistent with studies where olive oil extracts modulated cholesterol biosynthetic pathways in cell cultures by affecting ACACA and HMGCR activity (Lammi et al., 2020; Gnoni et al., 2021).

Both diets, DOP and WCOC, seem to have a significant impact on inflammatory and immune responses. In fact, high-fiber diets improve inflammatory responses (He et al., 2018) and alleviate oxidative stress (Tan et al., 2016) by changes in microbiota composition. In contrast, diets rich in SFA are associated with an increase inflammation markers (Karasawa et al., 2018). Adiponectin, which was activated in both experimental diets, is also involved in these functions, as it exerts anti-inflammatory effects by modulating several pathways, including the inhibition of foam cell formation, toll-like receptor-mediated NF-κB activation, reduction in the production of proinflammatory cytokines, and stimulation of anti-inflammatory factors (Ouchi and Walsh, 2007). Moreover, the DOP group showed inhibition in the expression of some cytokine genes, such as *Interleukin 7* (*IL7*) and *Complement C3* (*C3*) genes. The IL7 is a cytokine that stimulates the development of precursor cells for B and T lymphocytes, while C3 plays a pivotal role in activating the complement system (Copenhaver et al., 2019), crucial for regulating inflammation. Elevated levels of C3 have been associated with obesity and metabolic syndrome (Copenhaver et al., 2019). On the other hand, genes encoding anti-inflammatory factors such as *Pentraxin 3* (*PTX3*) were found to be activated in the DOP diet. Elevated concentrations of PTX3 have been associated with an induction of IL10 production, enhancing the anti-inflammatory response in the body (Slusher et al., 2016). This diet also showed increased expression of the *Epidermal Growth Factor* (*EGF*) gene, encoding a signalling protein that regulates cellular growth and differentiation, improving innate immune defence and inhibiting overactivation of pro-inflammatory functions in keratinocytes (Pastore et al., 2008). On the other hand, the DOP diet inhibited genes like *C-Type Lectin Domain Family 5 and 7* (*CLEC5A* and *CLEC7A*) which are involved in cell adhesion, cell-cell signalling, glycoprotein turnover, and roles in inflammation and immune response, and *Toll-Like Receptor 8* and *9* (*TLR8* and *TLR9*), which regulate inflammatory reactions and immune responses (Delneste et al., 2007). Additionally, the *Triggering Receptor Expressed on Myeloid Cells 2* (*TREM2*), crucial for promoting phagocytosis in microglia (Hsieh et al., 2009), was also inhibited. Similar results regarding genes belonging to these families, such as *TREM1*, *TLRs 2, 4, 6* and *8*, and *CLEC7*, have been observed in studies where a hydroxytyrosol rich diet reduced their expression (Laviano et al., 2024).

Similarly to DOP, in the WCOC group, the *CLEC5A, CLEC7A, TLR8, TLR9,* and *TREM2* genes were inhibited. Moreover, the *Leptin Receptor (LEPR*) and *Free Fatty Acid Receptor 4 (FFAR4*) genes were overexpressed in WCOC diet. Leptin is a hormone, primarily produced in adipose tissue, which regulates metabolism and appetite. Increased body fat leads to higher leptin levels, which suppress appetite and increase energy expenditure to maintain energy balance and prevent fat accumulation (Jéquier, 2002). Studies in Iberian pigs, knowing for their high food intake capacity (Morales et al., 2002), showed higher levels of circulating leptin compared to leaner breeds (Palma-Granados et al., 2017). A mutation in the *LEPR* gene linked to lower expression might explain a leptin resistant state associated with their greater feed intake (Óvilo et al., 2010). This aligns with the results observed in the WCOC pigs, which exhibited higher feed intake despite their higher expression of *LEPR* compared to the C group. FFAR4, a G protein-coupled receptor primarily expressed in the adipose tissue and immune cells, is activated by certain fatty acids, especially omega-3 fatty acids. This activation is associated with anti-inflammatory effects, inhibiting the release of proinflammatory cytokines such as TNF-α and interleukin-6 (Oh et al., 2010). In addition, FFA4 also regulates appetite and insulin sensitivity (Im, 2018). Its activation by omega-3 fatty acids agrees with the higher linolenic acid (C18:3) content in the WCOC diets (Supplementary Table S1), and the increased consumption of this diet, provided *ad libitum* in our study. In addition to the *Toll-Like Receptors* mentioned before, *TLR2* gene was also suppressed in the WCOC diet, along with other genes coding proinflammatory molecules such as *TNF Superfamily Member 8* gene (*TNFSF8*). Suppression of *TLR2* gene leads to the inhibition of proinflammatory transcription factors, such as NF-κB, reducing the production of proinflammatory cytokines and chemokines (Kawai and Akira, 2007). Our findings are supported by studies in rat, where supplementation with hydroxytyrosol, inhibited *TLR2* expression (Kawai and Akira, 2007). TNFSF8, a transmembrane protein expressed in immune cells, plays a key role in regulating the immune system by activating T lymphocytes, B cells, and dendritic cells upon binding to its receptor CD30 (Cerutti et al., 2000).

The potential effect of the diets on immune and immunological responses was also confirmed by several canonical pathways. Notably, both diets inhibited the *Phagosome Formation* pathway, a key mechanism in the innate immune response, allowing phagocytic cells to recognize, capture, degrade, and eliminate pathogens (Fountain et al., 2021). This inhibition was reflected by the downregulation of genes involved in recognizing and internalizing foreign particles, such as *Integrin Subunit Alpha M* (*ITGAM*), *Integrin Subunit Beta 2* (*ITGB2)*, and *Macrophage Scavenger Receptor 1* (*MSR1*). The activation of phagosomes in the adipose tissue of obese individuals is indicative of metabolic dysfunction, along with increased proinflammatory cytokines (Ju et al., 2019). Moreover, the *Phagosome Formation* pathway is closely linked to genes involved in the reactive oxygen species (ROS) production, indicating the connection between oxidation and inflammation. For instance, in the DOP diet, the reduced expression levels of *Cytochrome b-245* (*CYBB*) and *Neutrophil cytosolic factor 2* (*NCF2*) illustrate this connection (Giardino et al., 2017). Both CYBB and NCF2, which are components of the NADPH oxidase (NOX) complex responsible for generating ROS, play a role in the phagocytic activity. This suggests a decrease in NOX activity, potentially explaining the inhibition of the *Phagosome Formation pathway* (Table 3). Similarly, in the WCOC diet, a reduction in ROS production was observed again along with the inhibition of *CYBB* and *NADPH Oxidase Dual Oxidase 2* (*DUOX2*) genes. This decrease in ROS metabolism may be in part attributed to the reduction in lipid metabolic functions, leading to lower lipid oxidation.

The effect of the DOP diet on oxidative activity may also be associated with the relationship between the Apelin system and ROS (Figure 2). The inhibition of ROS generation is primarily mediated by mitogen-activated protein kinase (MAPK12) and AMPK, proteins encoded by genes overexpressed in DOP diet, which activate antioxidant enzymes such as superoxide dismutase, catalase, glutathione peroxidase 2 (*GPX2*, overexpressed in the DOP diet, Table 1), and NOX. Our results are consistent with studies highlighting the antioxidant properties of olive leaf extracts (Bhattacharjee et al., 2020; Rana et al., 2022). These antioxidants may contribute to blood pressure reduction by enhancing endothelial function, possibly by decreasing ROS levels (Bhattacharjee et al., 2020). Meanwhile, the protective function of polyphenols in eliminating ROS in the organism is well documented (Rana et al., 2022).

In the WCOC diet, additional pathways associated with inflammation were also inhibited, such as *Pyroptosis Signaling pathway*, *Pathogen-Induced Cytokine Storm Signaling pathway,* and *Role of Pattern Recognition Receptors in Recognition of Bacteria and Viruses*. Pyroptosis, an inflammatory programmed cell death, releases many pro-inflammatory factors, such as inflammasomes (Xue et al., 2019). The inhibition of pyroptosis may be attributed to the suppression of genes such as *Nucleotide-binding Oligomerization Domain, Leucine-Rich Repeat, Pyrin Domain-Containing 3* (*NLRP3*), and *PYD and CARD Domain Containing* (*PYCARD*), as well as various members of *TLR* genes. NLRP3 is crucial for inducing pyroptosis in response to various inflammatory stimuli and cellular damage (Coll et al., 2022), interacting with PYCARD protein, an essential adaptor protein of inflammasomes. TLR proteins and ROS from mitochondrial activity can activate the NLRP3 inflammasome (Kinra et al., 2022; Chung et al., 2020). The higher expression of the *NLRP*3 gene in the C group is in agreement with other studies showing that SFA-rich diets can activate the NLRP3 inflammasome in different tissues (Karasawa et al., 2018). Furthermore, olive derivatives’ impact on pyroptosis through these molecules has also been documented (Kaneko et al., 2019; Taticchi et al., 2019). Cytokines can also be induced by ROS (Mittal et al., 2014) causing severe inflammatory responses and tissue damage when excessively released, known as a cytokine storm (Karki and Kanneganti, 2021). The inhibition of *Cytokine Storm Signalling* in the WCOC diet may result from the downregulation of genes encoding chemokine ligands (e.g., *CXCL10*, *CXCL14*, and *CCL11*) and interleukins (e.g., *IL17D*, *IL27RA*, and *IL2RG*).

Finally, the WCOC diet induced apoptosis, or programmed cell death. This genetically regulated process involves the selective elimination of cells and may be attributed to the activation of caspases (Han et al., 2009). In this context, several genes that inhibit caspases, such as *B-cell lymphoma 2 Related Protein A1* (*BCL2A1*)*, Baculoviral IAP Repeat Containing 5* (*BIRC5*), and *Cyclin Dependent Kinase Inhibitor 1A* (*CDKN1A*), were downregulated in the WCOC. Several studies have reported the anti-proliferative and pro-apoptotic effects of olive oil and its derivatives, particularly relevant in the elimination of tumor cells (Isik et al., 2012).

The Iberian is an obese pig breed characterized by its high fat deposition and genetic predisposition to a proinflammatory status (Óvilo et al., 2010). In obese individuals, series of metaflammation processes occur in adipose tissue. This involves excessive lipid accumulation and oxidative stress, leading to chronic inflammation and the onset of various conditions such as insulin resistance and metabolic diseases. Imbalances in lipid metabolism result in an excess of free fatty acids, which can, in turn, increase oxidation reactions, thus generating an excess of ROS. Consequently, there is a closely related relationship between lipid metabolism, oxidative processes, and the immune system. The results described in this study revealed that both experimental diets based on olive by-products seem to alleviate inflammatory and oxidative processes in the animal, potentially modulated by different genes involved in different metabolic pathways. This effect could potentially improve animal welfare. However, experiments with individually controlled feeding would be recommended to elucidate the precise mechanisms and effects of the different dietary components on gene expression and metabolic regulation. Additionally, investigating physiological parameters such as metabolites, enzymes, and immunological compounds would greatly enhance our understanding of these complex interactions and their implications for overall health.

## Data Availability

The datasets presented in this study can be found in online repositories. Array data generated in this study were deposited into the Gene Expression Omnibus database (accession number: GSE264195).

## Abbreviations

ADFI, average daily feed intake; C, control; C_CF, compound feed of C; DOP, dry olive pulp; DOP_CF, compound feed of DOP; PUFA, polyunsaturated fatty acids; ROS, reactive oxygen species; SFA, saturated fatty acids; WCOC, wet crude olive cake; WCOC_CF, compound feed of WCOC.
